# Metabolomics of reef benthic interactions reveals a bioactive lipid involved in coral defence

**DOI:** 10.1098/rspb.2016.0469

**Published:** 2016-04-27

**Authors:** Robert A. Quinn, Mark J. A. Vermeij, Aaron C. Hartmann, Ines Galtier d'Auriac, Sean Benler, Andreas Haas, Steven D. Quistad, Yan Wei Lim, Mark Little, Stuart Sandin, Jennifer E. Smith, Pieter C. Dorrestein, Forest Rohwer

**Affiliations:** 1 Biology Department, San Diego State University, San Diego, CA, USA; 2 Collaborative Mass Spectrometry Innovation Center, Skaggs School of Pharmacy and Pharmaceutical Sciences, University of California at San Diego, La Jolla, CA, USA; 3 Carmabi Foundation, Piscaderabaai, Willemstad, Curaçao; 4 Aquatic Microbiology, Institute for Biodiversity and Ecosystem Dynamics (IBED), University of Amsterdam, Amsterdam, The Netherlands; 5 National Museum of Natural History, Smithsonian Institution, Washington, DC, USA; 6 Scripps Institution of Oceanography, University of California at San Diego, La Jolla, CA, USA

**Keywords:** metabolomics, coral, platelet activating factor, mass spectrometry

## Abstract

Holobionts are assemblages of microbial symbionts and their macrobial host. As extant representatives of some of the oldest macro-organisms, corals and algae are important for understanding how holobionts develop and interact with one another. Using untargeted metabolomics, we show that non-self interactions altered the coral metabolome more than self-interactions (i.e. different or same genus, respectively). Platelet activating factor (PAF) and Lyso-PAF, central inflammatory modulators in mammals, were major lipid components of the coral holobionts. When corals were damaged during competitive interactions with algae, PAF increased along with expression of the gene encoding Lyso-PAF acetyltransferase; the protein responsible for converting Lyso-PAF to PAF. This shows that self and non-self recognition among some of the oldest extant holobionts involve bioactive lipids identical to those in highly derived taxa like humans. This further strengthens the hypothesis that major players of the immune response evolved during the pre-Cambrian.

## 1. Introduction

The cellular recognition of self versus non-self is one of the most important biological processes. All organisms must interact and recognize others in their environment to elicit an appropriate cellular response. At the macro-organismal level, metazoan cells must exclude invaders (e.g. pathogens), control cheaters (e.g. cancerous growths) and select specific viruses [1] and microbes as part of their microbiome [2]. These assemblages of macrobes, microbes and viruses form an ecological unit known as a holobiont [3,4]. Reef building corals are a complex holobiont containing a spectrum of symbiotic associations from the obligately symbiotic alga *Symbiodinium* to thousands of stable and sporadically associated microbiota [5]. Corals competing with other reef holobionts for space on the benthos creates a mosaic of interactions [6,7], making coral reefs an ideal setting to study the evolution of self versus non-self recognition.

Cnidarian-like fossils appear in the Ediacaran Period (*ca* 635–542 Ma) [8]), placing them among the first animals on the planet. Evidence is accumulating that much of the mammalian immune system is also present in cnidarians, pushing its date of origin back to their last common ancestor living during the Pre-Cambrian (*ca* 550 Ma) [9]. Genes for many human immune proteins have been identified in cnidarian genomes and transcriptomes [10–13]. For example, toll-like receptors have been identified in the ancestor of all metazoans [14] and tumour necrosis factor alpha (TNFα), a central cytokine in both innate and acquired immunity, is functionally conserved in corals and humans [15]. Furthermore, a recently discovered adaptive immune response based on bacteriophage adherence to mucus, also occurs across these two distantly related Metazoa [1].

The inflammatory response is a fundamental cellular defence mechanism believed to have origins during the advent of multicellularity [16]. A hallmark of inflammation is the recruitment of phagocytic cells to sites of tissue injury or infection and this inflammatory-like response has been documented in corals [17]. Inflammation is a ubiquitous phenomenon among the Metazoa, presenting in a wide variety of forms, including melanization and phagocytosis at sites of infection in invertebrates [18] and phagocyte recruitment in anthozoans [19]. Despite this remarkable conservation, the signalling mechanisms of inflammation in basal metazoans are poorly understood compared with higher forms. Inflammatory lipid mediators such as prostaglandins [20,21], arachadonic acid metabolites [22] and eicosanoids [23,24] have been identified in corals, but little is known about the dynamics and conservation of function of these signalling molecules.

Algae are commonly found interacting with corals within reef communities around the world. Macro- and filamentous algae facilitate microbially mediated diseases [25], transfer of harmful molecules [26], and directly abrade and shade corals [27]. Transitions from coral to algal-dominated communities indicate reef degradation and are often driven by the D^3^AM dynamics (DOC, disease, direct contact, algae and microbes), where algae release dissolved organic carbon into the water column feeding microbial pathogens of corals [28–30]. As corals die, space is freed for algal colonization and growth, establishing a positive feedback loop for coral reef decline [31]. In addition, harmful hydrophobic compounds produced by algae can be directly transferred to corals [32], causing necrosis and/or apoptosis of coral tissue [26,33].

One challenge to using coral reefs to study immunological responses is that these ecosystems have been dramatically altered by anthropogenic stressors [34]. While pristine reefs remain dominated by stony corals (order: Scleractinia) and crustose coralline algae (CCA) [35], many modern reefs have shifted from coral to fleshy algal-domination [36,37], due to overfishing, pollution and climate change [31,36,38]. Such stressors may compromise effective immune responses of corals rendering them unable to outcompete neighbouring holobionts. To study the natural state of holobiont immune responses and interactions, it is therefore necessary to study them on unpopulated and pristine reefs that best reflect the historical dynamics of these threatened systems.

In this study, an untargeted metabolomics approach was used to analyse competing coral and algal holobionts in the Southern Line Islands (SLIs), one of the most remote and pristine coral reef systems remaining in the world [35]. Coral–algal interactions in the SLIs best approximate historical immune system responses of reef holobionts, because they have been minimally affected by anthropogenic influences. We observed corals responding to non-self interactions through alteration of their metabolomes and found that platelet activating factor (PAF), a known pro-inflammatory signal in human immunity, was a significant component of the coral metabolome and altered during competition.

## 2. Results

### (a) Holobiont metabolomic diversity

**Figure 1.**
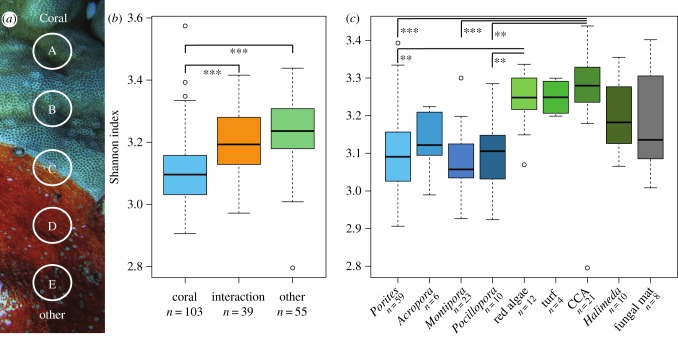
(*a*) An example of the interaction zone between a coral and a red alga that depicts the A, B, C, D and E sampling approach. (*b*) Shannon diversity indices of the metabolomes of corals, the interaction zone between corals and non-corals, and non-coral organisms. Statistical significance was determined using the Tukey's test of a one-way analysis of variance. *p < 0.05, **p < 0.01, ***p < 0.001. (*c*) Shannon diversity indices of metabolomes of organisms shown to a finer taxonomic level than in (*b*).

Tissue punches were collected across transects perpendicular to the interfaces of interacting holobionts (figure 1*a*). These interactions were classified as either coral:coral or coral:non-coral. Sampled coral holobionts included the genera *Porites*, *Montipora, Acropora* and *Pocillopora*. Non-coral holobionts included CCA (order: Corallinales), turf algae (mixed algal consortia), red macroalgae (*Peyssonnelia* spp.), calcareous green algae (*Halimeda* spp.) and a fungal mat from the Lulworthiaceae family (Ascomycota) as determined from 18S rDNA sequencing (a.k.a., Black Nasty; J. Janouskovec and F. Rohwer, 2015 unpublished data; sample details in electronic supplementary material, table S1). Five samples were collected from each holobiont–holobiont interaction (A, B, C, D, E sampling scheme, figure 1*a*).

Metabolomic diversity (Shannon's index) was higher in non-coral (3.24 ± 0.11) than coral holobionts (3.10 ± 0.11; Tukey's HSD test of an ANOVA, *p* < 0.001; figure 1*b*). Metabolomic diversity was also higher at the interaction zone (3.20 ± 0.10; *N* = 42; *p* < 0.001) compared with coral surfaces away from the interactions zone (figure 1*b*). The highest molecular diversity was found for CCA (3.27 ± 0.13), followed by turf algae (3.25 ± 0.04), *Peyssonnelia* (3.24 ± 0.07) and *Halimeda* (3.19 ± 0.09; figure 1*c*).

### (b) Self and non-self molecular relationships of holobionts

**Figure 2.**
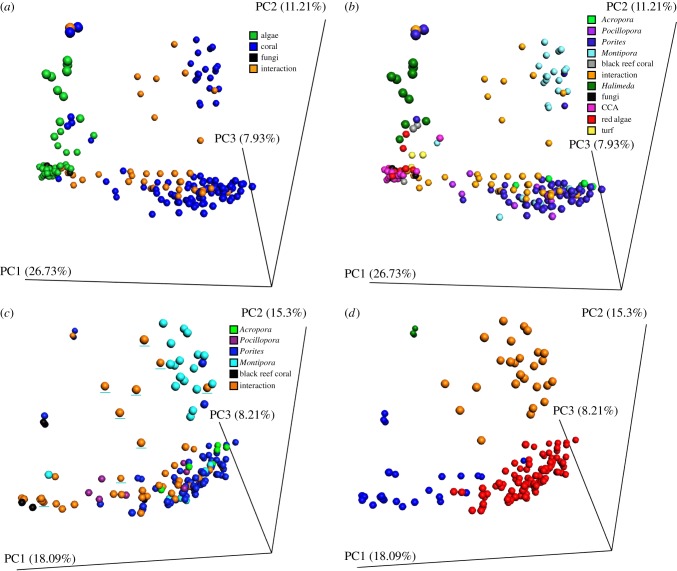
Holobiont metabolome relationships as determined by compressing the Bray–Curtis distance between samples into three-dimensional PCoA projections. (*a*) The entire SLI holobiont dataset, where samples are coloured by their category of organism (coral, non-coral or interaction). (*b*) The same dataset and projection coloured by the specific holobiont each sample represents. (*c*) Projection of the Bray–Curtis distance of only the coral and their interaction samples coloured by the specific holobiont each sample represents. The *Montipora* interaction samples are underlined with a teal line to highlight them from the others. (*d*) The same PCoA projection of the coral Bray–Curtis distance coloured by the four hierarchical clusters identified using silhouettes. The per cent of variance explained by each principle coordinate is shown on the axis.

Principle coordinate analysis (PCoA) of the metabolomic data from all samples separated non-coral and coral with the interaction samples in between (figure 2*a,b*). The clustering of coral, algae and interaction samples (PERMANOVA *F* = 16.58, *p* = 0.001, figure 2*a*) and a deeper classification based on specific organism/genera (PERMANOVA *F* = 10.51, *p* = 0.001, figure 2*b*) were both significant. Visualization of sample types with genus-level resolution showed that one of the coral clusters consisted mainly of samples taken from *Montipora* spp. (100% of cluster *Montipora* or *Montipora* interaction samples), with all other coral genera forming the other cluster (figure 2*b*). A similar pattern was observed with the non-coral metabolomes, where samples from *Halimeda* spp. were separated from a tight group of all other non-coral samples. Coral samples classified based on their distance from the interaction zone (A, B or D, E) did not produce significant clustering (sample location PERMANOVA *F* = 2.17, *p* > 0.05), however, there was a slight effect of island for the *Porites* only data (PERMANOVA *F* = 2.67, *p* = 0.001).

Metabolomes analysed from four samples taken from corals on a ‘black reef’, unique coral reef ecosystems caused by iron enrichment associated with shipwrecks in marine areas naturally depleted in iron [39], were different from all other coral samples and clustered with the algal samples (figure 2*b*). Hexadellin A (*m/z* 713.837, electronic supplementary material, figure S1) was putatively identified in a black reef coral, but no other coral samples.

Analysis of the coral samples alone confirmed that *Montipora* spp. metabolomes and their interaction samples were different from all other coral genera (PERMAVONA *F* = 6.31, *p* = 0.001, figure 2*c*). The silhouette plot of the hierarchical clustering analysis (HCA) indicated that there were four distinct clusters (electronic supplementary material, figure S2) containing: (i) *Montipora* samples (94% of cluster *Montipora* or its interaction samples), (ii) other corals samples (72% non-*Montipora* coral), (iii) a mix of interaction samples and other corals, and (iv) a cluster of three *Porites* samples that were interacting with a Black Nasty fungal mat (ascomycete family Lulworthiaceae) (figure 2*d*). This smaller fourth cluster indicated that the Black Nasty interaction dramatically affected the *Porites* coral in this instance. The closely related corals *Acropora* and *Montipora* [40] also had significantly different metabolomes (*Acropora* versus *Montipora* PERMANOVA *F* = 5.37, *p* = 0.002). Similar to the corals, separate statistical analysis of the non-coral holobiont metabolomes (samples D, E) verified the unique nature of samples of the genus *Halimeda* (non-coral classification PERMANOVA *F* = 5.79, *p* = 0.001, electronic supplementary material, figure S3). The *Halimeda* samples comprised their own unique cluster (92% *Halimeda*), while the other non-corals samples clustered together (electronic supplementary material, figure S3).

### (c) The influence of self and non-self interactions on coral metabolomes

Unique MS/MS spectra (a proxy for molecules) of the entire SLIs dataset were identified using a molecular networking algorithm [41]. There were 33 634 unique spectra of which 8230 (24.5%) were detected in coral samples only, 5412 (16.4%) only in the interaction samples (C sample) and 5684 (16.9%) only in non-coral samples (D or E samples, electronic supplementary material, figure S4). Only 2448 (7.3%) spectra were detected in all sample types.

**Figure 3.**
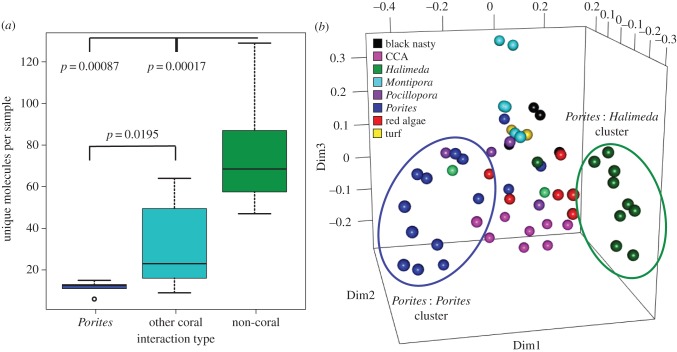
Effects of self versus non-self competition on the *Porites* metabolome. (*a*) Boxplots of the distributions of the number of unique molecules per sample from the adjacent molecular network calculated by interaction group. (*b*) MDS plot of a supervised random forests of the top 30 most variable molecules when *Porites* was interacting with a self or non-self holobiont. Clusters of *Porites* interacting with *Porites* and *Porites* interacting with *Halimeda* are highlighted.

Owing to the large number of *Porites* spp. samples (*n* = 59), specific metabolomic changes at its interaction sites could be analysed in detail. Out of a total of 4382 unique molecules in the *Porites* dataset, 1107 were unique to interactions between *Porites* spp. and non-coral holobionts, 163 to *Porites* spp. interacting with other coral holobionts and 44 to *Porites* holobionts interacting with each other. More unique molecules were produced when *Porites* holobionts interacted with non-coral holobionts (Wilcoxon's rank-sum test, *Porites* versus other coral *p* = 0.020, *Porites* versus non-coral *p* = 0.00087; figure 3*a*). Clustering analysis of the *Porites* data revealed a *Porites–Porites* cluster, a *Porites–Halimeda* cluster, and two intermixed clusters of all other interactions. These separate clusters indicated that changes in *Porites*' metabolomes were largest when it competed with *Halimeda* spp. (figure 3*b*). Thus, metabolomes of *Porites* changed in interactions with non-*Porites* neighbours, especially when interacting with *Halimeda* spp., but remained largely unchanged when *Porites* colonies were involved in self-interactions.

### (d) Lyso-platelet activating factor and platelet activating factor in the coral metabolome

The 30 most differentially abundant molecules between coral and non-coral were identified using the variable importance plot (VIP) feature of the supervised random forests algorithm. Two very abundant molecules only found in corals were putatively classified as Lyso-PAF-C:18 (*m/z* 510.42) and Lyso-PAF-C:16 (*m/z* 482.36; electronic supplementary material, figure S5) after a parent mass search of the METLIN database [42]. These molecules were subsequently verified through a GNPS library search and a purchased Lyso-PAF-C:16 standard (Sigma-Aldrich^®^, St Louis, MO, USA; electronic supplementary material, figure S6).

**Figure 4.**
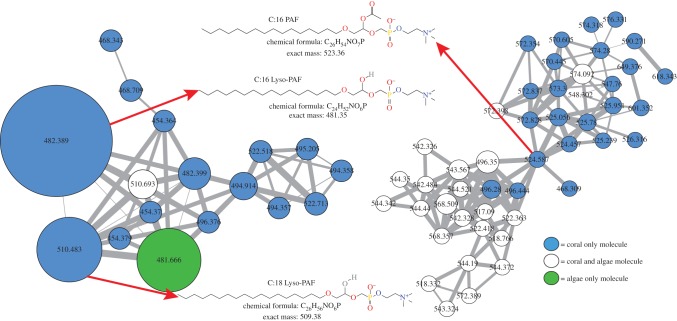
(*a*) Lyso-PAF/PAF molecular clusters as identified by molecular networking and putative structures of known molecules. Node sizes are scaled to the total abundance in the entire metabolome and coloured based on holobiont source. Edges widths are scaled to the cosine score.

The identification of Lyso-PAF led to a search for PAF, the acetylated molecule, which was found in C:16 and C:18 forms in coral (figure 4). The C:16 form was present in all coral metabolomes (99/99 coral samples). The relative abundance of Lyso-PAF-C:16, Lyso-PAF-C:18, PAF-C:16 and PAF-C:18 was subsequently measured for all coral samples (electronic supplementary material, figure S7 and table S2). Lyso-PAF-C:16 was the most abundant molecule in the entire coral metabolome and absent from all non-coral samples (electronic supplementary material, figure S7 and table S2). PAF, the active form, was less abundant than Lyso-PAF, and also highly prevalent in coral samples (96/99 coral samples) and absent in all non-coral samples. The C:18 form of PAF was only occasionally detected in corals (14/99 samples) and was of very low abundance.

Several other molecules were putatively annotated through GNPS library searching of the SLI data (electronic supplementary material, table S3). The fatty acid *cis*-7,10,13,16,19-docosapentaenoic acid, a common component of fish oils, was absent from all non-coral samples and significantly more abundant in *Acropora* coral than all other holobionts (Tukey's HSD of ANOVA *p* = 0.02). A related metabolite *cis*-4,7,10,13,16,19-docosahexaenoic acid was absent from *Montipora*, but commonly detected in *Porites* samples. Eicosapentanoyl ethanolamide, a known anti-inflammatory eicosanoid [43], was prevalent and abundant in coral samples except *Porites* (electronic supplementary material, table S3). This metabolite was abundant in *Montipora* and decreased in abundance towards the interaction zone (electronic supplementary material, figure S8, Pearson's *r* = 0.45, *p* = 0.006), but did not correlate to Lyso-PAF or PAF abundance, two molecules known as central inflammatory modulators.

### (e) Detection and expression of coral encoded genes of Lyso-platelet activating factor/platelet activating factor

**Figure 5.**
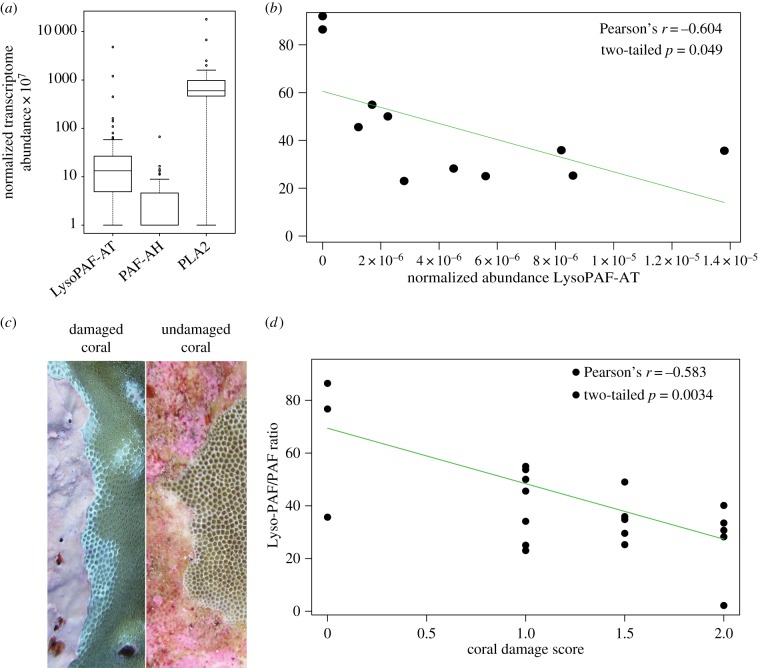
(*a*) Normalized transcriptome abundances of *Lyso-PAF-AT*, *PAF-AH* and *PLA2* in all transcriptome samples. (*b*) Regression of the Lyso-PAF/PAF ratio and abundance of *LysoPAF-AT* in the same coral transcriptome and metabolome interaction samples. (*c*) Images of a damaged and undamaged coral from the SLIs dataset. (*d*) Regression of the Lyso-PAF/PAF ratio compared with the increasing coral damage score in the interaction samples.

Owing to the ubiquity and abundance of Lyso-PAF and PAF in the coral metabolomes, we searched for the genes that interconvert Lyso-PAF and PAF in the published genome of the coral *Acropora digitifera* [11]. Lyso-PAF acetyltransferase (*LysoPAF-AT*), platelet activating factor acetylhydrolase (*PAF-AH*) and phospholipase A2 (*PLA2*) were all present in the *A. digitifera* genome (electronic supplementary material, table S4). Transcriptome data generated from the same samples used for the metabolomic analysis were analysed for the presence and abundance of *PLA2*, *LysoPAF-AT* and *PAF-AH* gene transcripts (*n* = 58 transcriptomes, electronic supplementary material, tables S1 and S5) and all three enzymes were present in the SLI transcriptome data (figure 5*a*). The most abundantly expressed gene was *PLA2* (mean normalized abundance 1.16 × 10^−4^ ± 2.4 × 10^−4^), followed by *LysoPAF-AT* (1.36 × 10^−5^ ± 4.5 × 10^−5^). *PAF-AH* had the lowest expression (4.4 × 10^−7^ ± 9.9 × 10^−7^) and was detected in 70% of the coral samples (figure 5*a*).

The Lyso-PAF/PAF abundance ratio was compared with the expression level of genes involved in the interconversion of these two lipids, to identify a correlation between the transcripts and metabolites involved in this response in corals. In the paired transcriptome/metabolome interaction samples, the Lyso-PAF/PAF ratio was regressed against the normalized abundance of *LysoPAF-AT* and *PAF-AH*. There was no relationship between the abundance of *PAF-AH* gene transcripts and the Lyso-PAF/PAF ratio (two-tailed of Pearson's *r*, *p* > 0.05), but a negative relationship existed between the expression of *LysoPAF-AT* (Pearson's *r* = −0.604, *p* = 0.049; figure 5*b*). To test the hypothesis that coral holobionts were converting Lyso-PAF and PAF in response to competition with other benthic holobionts, the Lyso-PAF/PAF ratio was compared with the degree of tissue damage in *Porites* interaction samples. The Lyso-PAF/PAF ratio decreased with increasing coral tissue damage (figure 5*c,d*; Pearson's *r* = −0.583, *p* = 0.003).

## 3. Discussion

A metabolomics and transcriptomics approach was used to investigate self versus non-self interactions among benthic organisms on pristine coral reefs. Non-self competition significantly altered coral metabolomes. At the interaction zone, more than 5000 unique molecules were detected, indicating a specific chemical signature to competition among the holobionts. Furthermore, *Porites* holobionts responded more strongly to competition with non-self than self. Some macro- and turf algae promote microbial growth resulting in anoxia on neighbouring corals because of the release of dissolved organic carbon [25,29,44,45]. This stress mechanism induced by algal competitors could be responsible for the altered metabolomes of corals observed here. The *Porites* metabolome was altered most in response to the alga *Halimeda*. This calcifying macroalga has been shown to induce disease upon contact with corals in the Caribbean [46] and contribute to hypoxia at the interaction interface [29]. Direct contact with *Halimeda* may also alter the coral metabolome through the transfer of secondary metabolites. Some fleshy macroalgal species produce hydrophobic molecules that harm coral [33,47]. However, the specific terpenes previously identified by [32] were not detected in any of our samples.

### (a) Metabolomic relationships among reef holobionts

In general, the metabolome composition differed predictably among taxa, with the non-coral samples being distinct from coral and interaction samples representing a mix of both types. However, the metabolomes of *Montipora* differed from the other three coral genera, *Pocillopora*, *Porites* and *Acropora.* Metabolomic differences among coral taxa have been observed previously [48], supporting these findings. The metabolomic relationships within the corals did not mirror evolutionary relationships. For example, while *Acropora* is closely related to *Montipora*, both members of the family Acroporidae [40], their metabolomes were significantly different (figure 2*c*). Therefore, metabolomic variation may reflect differences in ecological characteristics of coral holobionts (e.g. growth, morphology, life-history strategy and/or microbiome) instead of evolutionary relationships.

Specific metabolites that differentiated corals included eicosapentanoyl ethanolamide and *cis*-4,7,10,13,16,19-docosahexaenoic acid. The former molecule is potentially involved in interactions because of its decreased abundance towards the interaction zone in *Montipora*. This metabolite has been shown to be anti-inflammatory [43], and its decreased production at the interaction interface supports this property in corals. However, expression of the AOS-LOXa enzyme, responsible for production of many eicosanoids, has been shown to be elevated adjacent to the site of coral wounding and increased with severity of the wound stress [24]. Thus, much like humans, the chemical specificity of signalling lipids is crucial to their biological activity; different eicosanoid chemical species may also induce contrasting responses in corals. The molecules identified here, and others previously detected in corals, such as prostaglandins [20,21], arachadonic acid metabolites [22] and eicosanoids [23,24], are all known to have roles in immunity. In this study, we show that coral genera have a varied abundance and prevalence of these lipids, indicating they may have different lipid metabolisms and/or signalling pathways.

Metabolomes of black reef corals were more similar to algae than to corals from pristine reefs (figure 2*c*). Black reefs are iron-induced phase shifts caused by shipwrecks leaching iron into otherwise iron-deplete reef systems. Black reefs are known to alter the microbial metagenome of the coral holobiont [39]; here we show it also drastically changes the metabolome. Hexadellin A, a natural product produced by the sponge *Hexadella* spp. [49], was identified only in corals on black reefs. This molecule has antibacterial activity against pathogenic organisms [50], potentially representing a cnidarian response to the increased pathogen load associated with black reefs [39]. Further studies into natural products uniquely produced on black reefs may provide insight into other aspects of the disease mechanism underlying iron enrichment.

### (b) Lyso-platelet activating factor and platelet activating factor in coral interactions

Corals, but not algae, contained various forms of Lyso-PAF and PAF. Lyso-PAF has previously been found in sponges [51] and corals [52], is produced in response to tissue damage in terrestrial invertebrates [53], deters fouling in sponges, [54], and has antimicrobial properties [55]. PAF is a single fatty acid chain phospholipid that acts as a potent signalling chemical to induce inflammation [56]. In humans, Lyso-PAF is converted to PAF by *LysoPAF-AT*, and in turn, back to Lyso-PAF, by *PAF-AH* [57]. Production of PAF activates neutrophils via NADPH oxidase leading to priming, production of elastase and superoxide, and interactions between platelets and neutrophils [58]. Upon conversion of PAF back to Lyso-PAF the inflammatory response is reduced, making the Lyso-PAF/PAF interconversion a molecular switch of inflammation in humans [57]. We provide evidence that this molecular switch is also present in corals, representing another evolutionarily conserved molecular response to stress induced by self and non-self interactions.

The Lyso-PAF/PAF ratio decreased with increased coral tissue damage, supporting the hypothesis that PAF is produced from Lyso-PAF in response to the tissue damage. The change in the Lyso-PAF/PAF ratio was most probably driven by the acetyltransferase activity of the coral homologue of *LysoPAF-AT*, which was detected in all available coral genomes. *LysoPAF-AT* expression was also negatively correlated with Lyso-PAF/PAF ratio in the metabolomic dataset, indicating this gene was expressed when PAF was relatively more abundant in damaged tissue. However, there was not a correlation between *PAF-AH* and this ratio, suggesting the switch back to Lyso-PAF may not be completely controlled by this enzyme. Inflammation and lipid signalling is complicated in many systems, often having pleiotropic effects, including that of PAF in humans [59]. Nevertheless, the metabolome and transcriptome data indicate that *LysoPAF-AT* and Lyso-PAF/PAF interconversions are important elements of the immune responses of basal metazoans. The classic lipid-remodelling pathway through lysophospholipid acetyltransferases is a well-studied physiological response to inflammation in higher eukaryotes [60]. These enzymes have been identified in a number of opisthokonts besides just the Metazoa, including the Protista [61], indicating acetyltransferase activity on lysophospholipids may have preceded the evolution of mutlicellularity. An expansion of these genes has been identified in both the Metazoa and Deuterostomia [61], signifying their functionality greatly expanded as eukaryotes became more complex multicellular organisms. This expansion may have been due to the development of lysophospholipid acetyltransferase activity and lipid remodelling as an immune signalling mechanism, such as that observed in this study.

## 4. Conclusion

**Figure 6.**
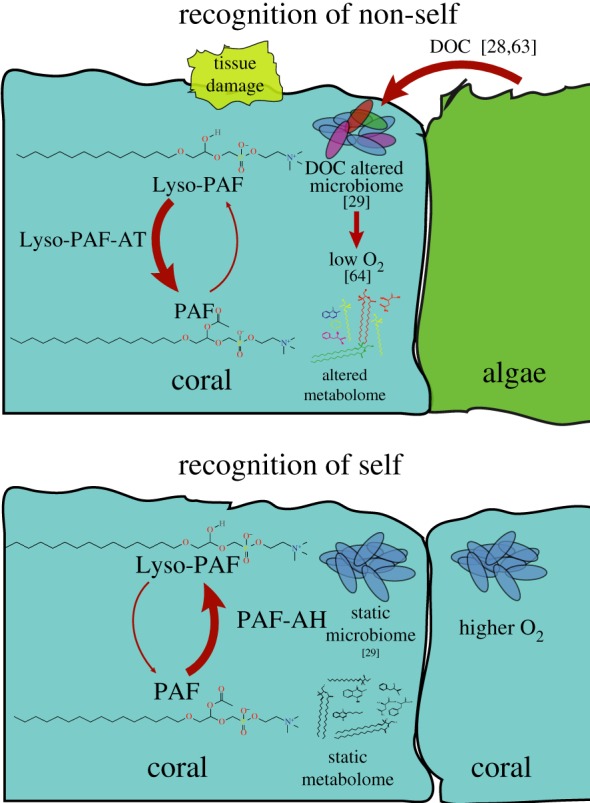
Model of Lyso-PAF and PAF response to non-self invasion in the coral holobiont.

We provide evidence that PAF can act as a signalling molecule in corals responding to encroaching organisms. In the proposed model, interaction with a non-self holobiont induces a multitude of changes at the interaction interface (figure 6). It has been previously shown that algal interactions alter the coral microbiome and induce tissue damage [29]. Evidence suggests that this alteration is due to the release of DOC from algal photosynthesis [28,30], which fuels microbial aerobic respiration on corals, drawing down oxygen levels [44,45]. This study demonstrates that algal and microbial induced tissue damage also changes the overall metabolome of the coral and results in the production of PAF from cells at the interaction interface where it potentially acts as a molecular signal of host response to this harmful encroachment. Thus, changes in the Lyso-PAF/PAF ratio in coral tissues may be analogous to its effect in humans where it induces inflammatory cascades [57], representing another highly conserved immune pathway through 550 Ma of metazoan evolution.

## 5. Material and methods

### (a) Sample collection and extraction

All samples were collected in October and November 2013 on five islands that are part of the SLIs: Flint (−11.43000°; −151.8192000°), Vostok (−10.100000°; −152.383333°), Malden (−4.020531°; −154.932059°), Starbuck (−5.641207°; −155.878208°) and Millenium (−9.936981°, −150.211500°). Five 1 cm wide discs were collected using punch chisels starting with the coral holobiont 5 cm and 1 cm away from the competitive interface (samples A and B), then an interaction sample in between (sample C), and lastly, two more samples (samples D and E) were collected 1 cm and 5 cm within the organism that corals competed with. Samples were placed into 10 ml of LC-MS/MS grade 70% methanol and 30% water for metabolite extraction.

### (b) Liquid chromatography-tandem mass spectrometry

Mass spectrometry was performed using a Bruker^®^ Daltonics Maxis qTOF mass spectrometer equipped with an electrospray ionization source. A water–acetonitrile gradient (from 98 : 2 to 2 : 98 water : acetonitrile, 0.1% formic acid) was used as the mobile phase. The flow rate was 0.5 ml min^−1^ and the mass spectrometer was operated in data-dependent positive ion mode, automatically switching between full scan MS and MS/MS acquisitions after every 10 MS/MS fragmentations. Automatic exclusion was used with parameters set such that an ion would be ignored if seen for three scans, but then refragmented if its intensity was 2.5× the previous scan. A Lyso-PAF-C16 standard was purchased from Sigma-Aldrich, diluted to 3 µM in ethanol and subjected to LC-MS/MS analysis according to the same parameters as outlined above.

### (c) Molecular feature table generation

The molecular features of each metabolome were called using the Bruker Daltonic Find Molecular Features (FMF) algorithm on each sample in a batch process using the Bruker DataAnalysis software v. 4.2 build 4.2.395.0. These were normalized to the total abundance of molecules detected in each sample and then the top 3500 most abundant molecules were used for further analysis. This matrix was imported to the R-Studio software package v. 0.97.318 for all statistical analysis.

### (d) Statistical analyses

Overall metabolomes were compared using the Bray–Curtis dissimilarity and then reduced to the top three principle coordinates for visualization. A PERMANOVA test was done to test for cluster significance with 999 permutations. The numbers of clusters were determined using silhouettes of a HCA. All statistical calculations were performed in R with the ‘vegan’ package. Further statistical analysis using the same methods was done on the coral samples (including their interaction ‘C’ samples) and the algae sample matrices separately to validate clustering observed on the entire dataset.

To identify how the various holobionts affected the *Porites* metabolome a supervised random forests was first run to identify the top 30 molecules that were most variable during different holobiont interactions in the A and B *Porites* data. These 30 molecules were used for an unsupervised random forests as a dimension reduction strategy to visualize specific sample relationships. PAM clustering and silhouette plots were used to identify statistically significant clusters. All random forests analysis was performed in R using the ‘randomForest’ package.

A supervised random forests, with samples identified as either coral or algae (interaction samples not included), was used to classify the samples based on the metabolomic data and identify variables of importance that best distinguished coral from algae. The VIP from the random forests using all coral samples (A and B) and all non-coral samples (D and E) was used to identify the molecules that best distinguished the two groups.

Shannon indices of metabolomic diversity were tested across holobionts using the Tukey's HSD test of an ANOVA. The distribution of the number of unique molecules per *Porites* sample based on molecular networking was tested for normality with a Shapiro–Wilk's test. As not all groups were normally distributed a two-tailed Wilcoxon's rank-sum test was used to test for significant differences in the pairwise comparisons of the three groups with a correction for multiple comparisons.

### (e) Molecular networking

Molecular networking was carried out as described in [41,62]. Molecular networks were generated using GNPS (gnps.ucsd.edu). Library searching for known molecules was done using the GNPS library search features. Molecular networking parameters for network generation and library searching are available in the electronic supplementary material. Comparing MS/MS spectra and LC retention times of the coral samples with a standard of Lyso-PAF verified the presence of this molecule.

### (f) Platelet activating factor statistics

Area under the curve Lyso-PAF and PAF abundances were calculated manually using Bruker DataAnalysis software and then the ratio of Lyso-PAF/PAF was used as to test for significant differences in the metadata. A comparison between the Lyso-PAF/PAF ratio was made between unaffected coral samples (designated with 0) and those that exhibited significant signs of damaged tissue at the interface (1 or 2). Corals were classified as slightly damaged (1) when coral polyps immediately bordering the interface were bleached, or showed clear signs of stress, or severely damaged (2) when larger regions of the colony were bleached, or showed clear signs of stress, or if deceased coral polyps were visible. A Pearson's correlation was run on the relationship between the Lyso-PAF/PAF ratio and coral damage score and tested for significance at a level of *p* < 0.05.

### (g) Bioinformatic identification platelet activating factor-related gene homologues in coral

BLASTp was used to search the translated genome of *A. digitifera* (available here: http://marinegenomics.oist.jp/genomes) with the human sequences of *PAF-AH* (GI 189095271), *LysoPAF-AT* (GI 126364244) and *PLA2* (GI 189953). Homologues of the human genes in the coral genome were selected from best hits, lowest *e*-value and highest per cent identities with the human query.

### (h) Metatranscriptome generation and analysis

Coral punches for transcriptomics were stored in RNAlater (Life Technologies) immediately after collection and then at −80°C. Total RNA was extracted from coral punches using the AllPrep DNA/RNA kit (Qiagen). RNA-seq libraries were constructed using Illumina TruSeq Stranded mRNA kit and sequenced at the UC Davis Genome Center using an Illumina HiSeq with 2 × 250 paired-end read chemistry. Raw reads were trimmed for sequencing adaptors and quality filtered using BaseSpace FastQ toolkit (Illumina) (electronic supplementary material, table S5).

Sequences of the genes of interest from the *A. digitifera* genome were used as a query for a BLAST search against the SLI transcriptomes as a database using tBLASTn in Geneious and an *e*-value cut-off of 10^−5^. Protein hit abundances were normalized to the total number of sequences per sample and then scaled to protein length (LysoPAF-AT = 544aa, PAF-AH = 440aa, PLA2 = 145aa). Comparisons between metabolome and transcriptome Lyso-PAF and PAF abundances were done on the interaction samples only.

## Supplementary Material

Supplemental Figures

## Supplementary Material

Supplementary Methods

## Supplementary Material

Supplementary table captions

## Supplementary Material

Table S1

## Supplementary Material

Table S2

## Supplementary Material

Table S3

## Supplementary Material

Table S4

## Supplementary Material

Table S5

## Data accessibility

The metabolomics data are available at gnps.ucsd.edu with MassIVE ID#MSV000078598.
