# Supplementary material for: Metabolomics of reef benthic interactions reveals a bioactive lipid involved in coral defence
Source: Proc Biol Sci. 2016 Apr 27;283(1829):20160469. doi: 10.1098/rspb.2016.0469 (PMC4855392; doi:10.1098/rspb.2016.0469)

Figure S1. A mirror plot of the spectral alignment of Hexadellin A and a query spectrum from a black reef coral sample. Query spectrum is black and the library spectrum is green. This alignment has a cosine score of 0.894, with 9 matched peaks and a mass difference of *m/z* 0.015.

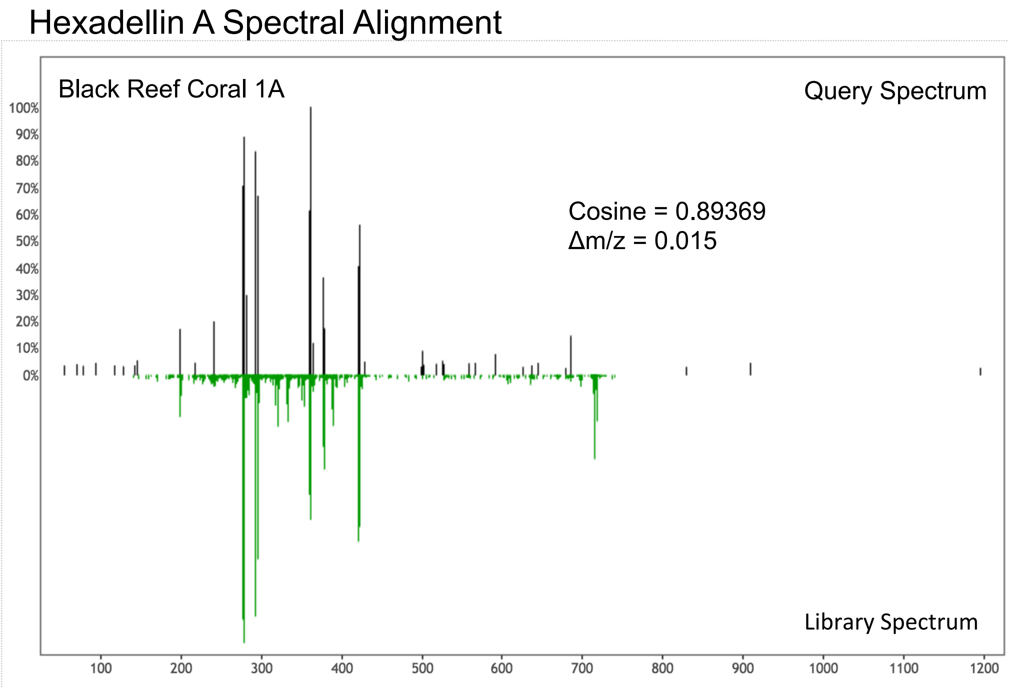

Figure S2. Silhouette plot of the strongest number of groups from the coral/interaction sample random forests. The peak indicates that the data contained the strongest clustering at k=3 groups.

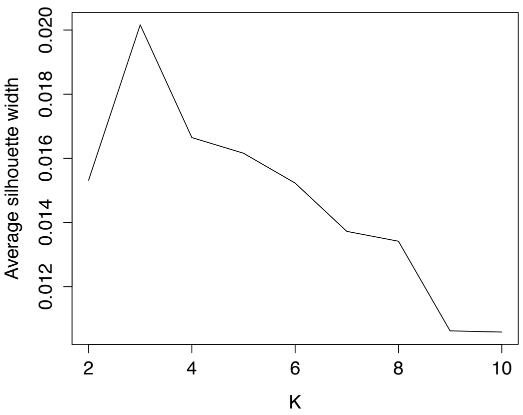

Figure S3. Principle Coordinates projection of a Bray-Curtis dissimilarity matrix produced on the non-coral metabolomic data.

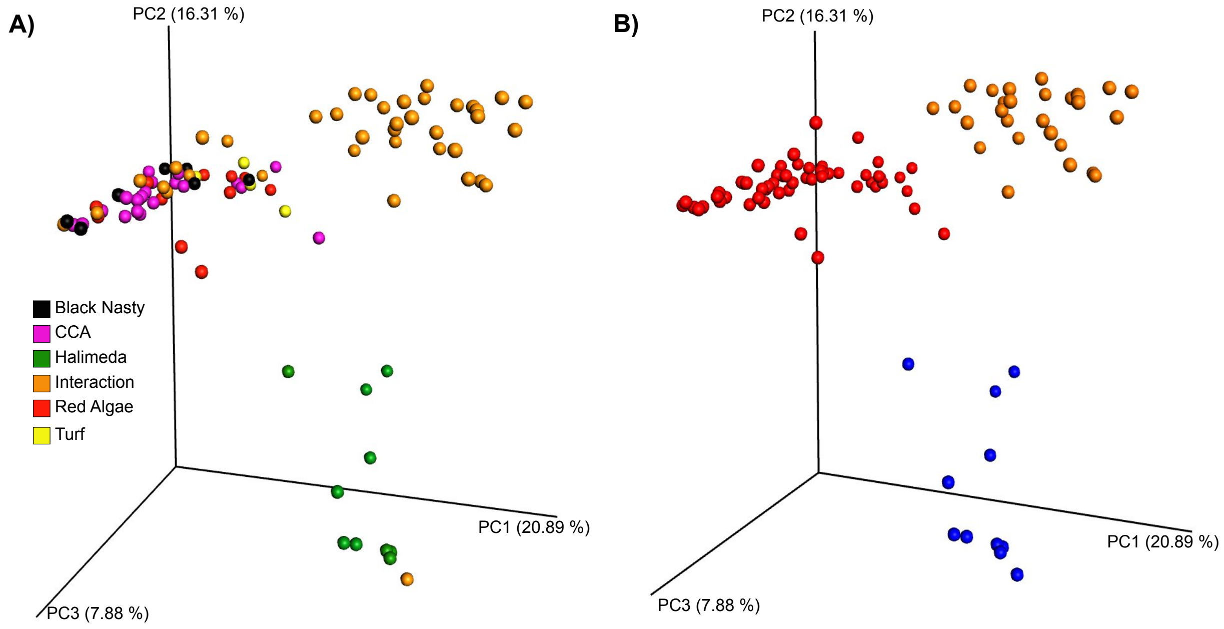

Figure S4. Venn diagram of the membership of unique spectra in the entire SLI metabolomics data set.

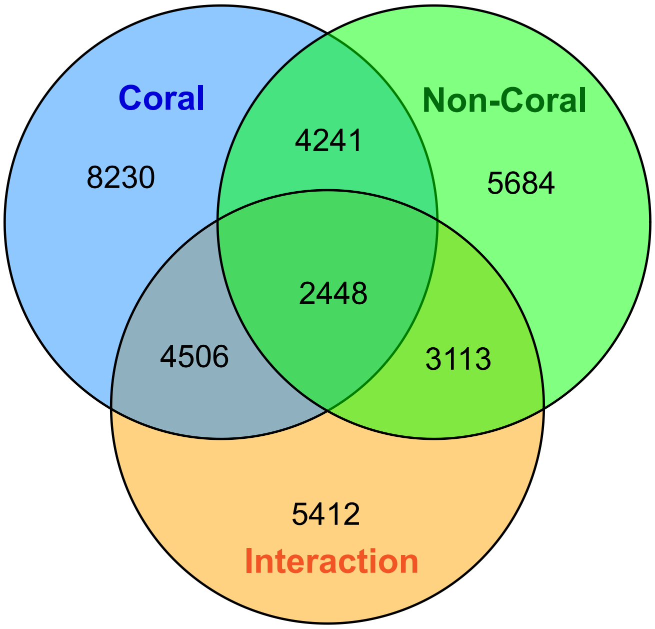

.

Figure S5. Variable importance plot of the coral versus algae random forests.

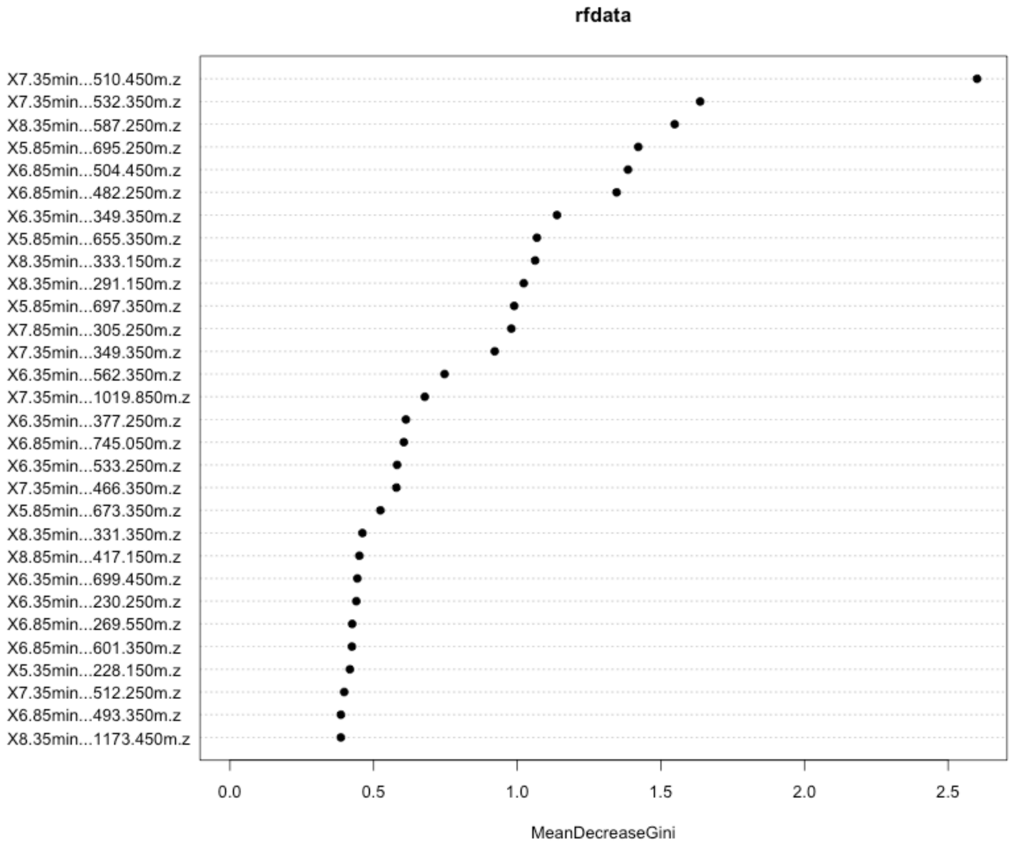

Figure S6. Molecular clusters of Lyso-PAF and PAF from purchased standards and a coral sample.

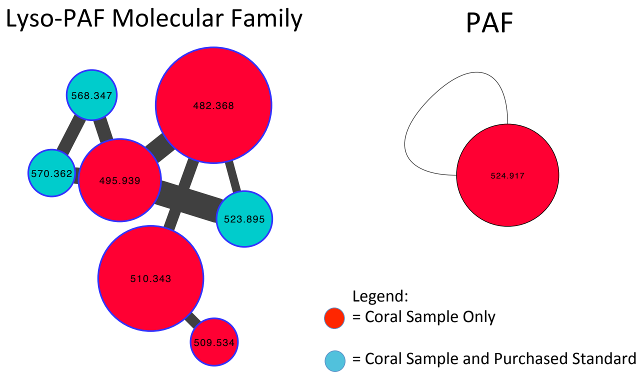

Figure S7. Normalized abundances of LysoPAF C:16, LysoPAF C:18, PAF C:16 and PAF C:18.

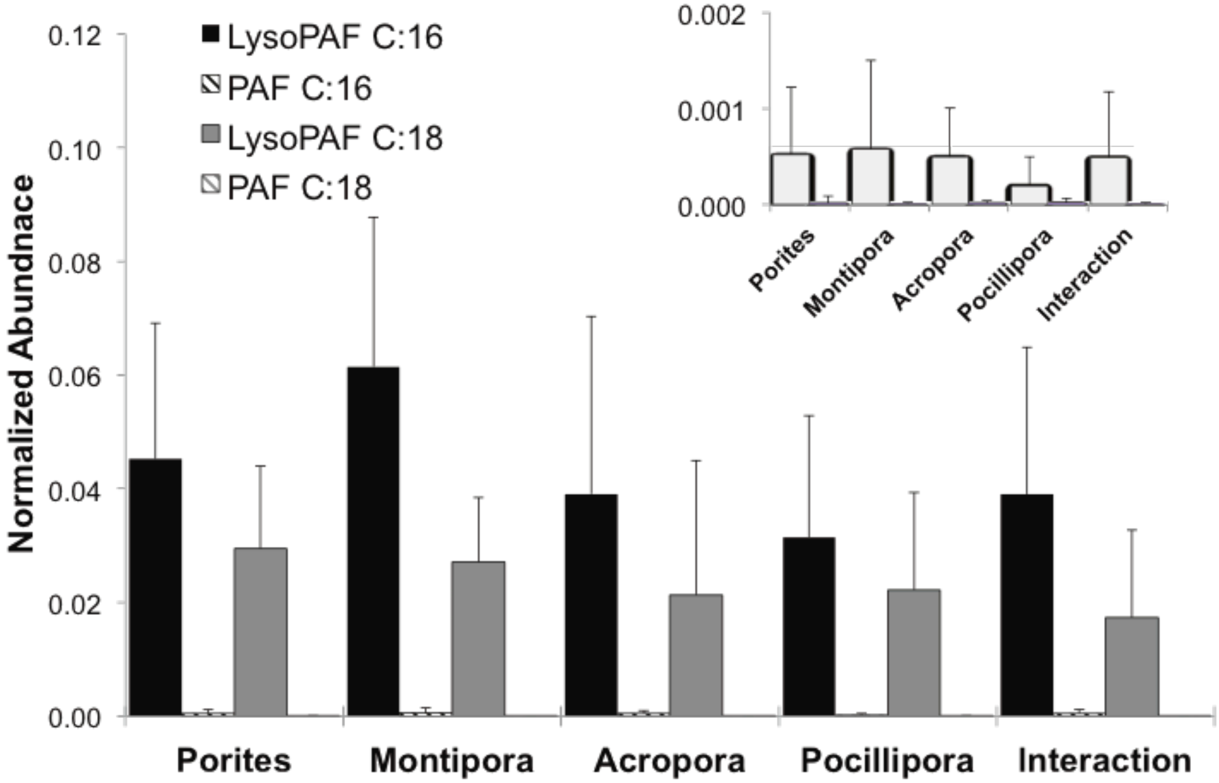

Figure S8. Normalized abundance of eicosapentanoyl ethanolamide in *Montipora* samples. The correlation between the distance from the interaction zone (in cm) and normalized abundance of the metabolite was significant (Pearson's r = 0.44, p = 0.006, A:C comparison is statistically significant according to a Tukey's test of a one-way ANOVA p=0.026).

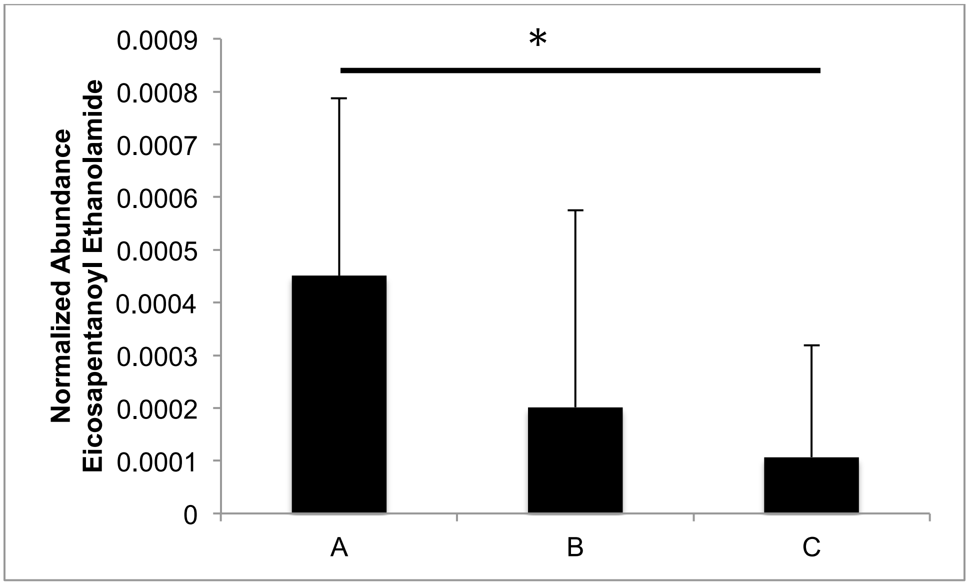

Supplement: Supplemental Figures [file rspb20160469supp1.pdf]
