# Supplementary material for: Metabolomics of reef benthic interactions reveals a bioactive lipid involved in coral defence
Source: Proc Biol Sci. 2016 Apr 27;283(1829):20160469. doi: 10.1098/rspb.2016.0469 (PMC4855392; doi:10.1098/rspb.2016.0469)
Supplement: Supplementary Methods [file rspb20160469supp2.docx]

**Supplementary Methods:**

*Methods overview:*

Punches (approximately 3 cm in diameter) were taken using a hammer and round chisel from interacting holobionts (Figure 1a). Compounds were extracted from these samples in 70% methanol and analyzed using LC-MS/MS. Metabolomic profiles were generated by calculating the abundances of molecular features identified with LC-MS and molecule identifications were performed using a MS/MS spectral alignment algorithm molecular networking [1] in combination with the Global Natural Product Social molecular networking (GNPS) online database (gnps.ucsd.edu). A supervised Random Forests was used to identify molecular features that significantly distinguished among the various sampled holobionts, while Bray-Curtis distance matrices and hierarchical clustering were used to compare and visualize sample relationships

*Sample collection and Extraction:* Samples were collected on the shallow fore reef (5-10m depths) at 2 to 3 sites per island whereby the most commonly occurring benthic interactions were targeted for collections (Table S1). The interaction zone was sampled so that both competing organisms were equally abundant (sample C, Figure 1a). Each sample was stored individually in plastic collection bags and brought to the boat within one hour after collection. Interaction samples included instances in which organisms were separated by no more than 2 mm, and in most cases included direct contact.

*Statistical Analysis*: All PCoA plots were visualized using the 3D emperor plot software [2]. Metabolomic comparisons were run on the entire 198 sample data set as well as the coral only and non-coral only subsets.

*LC-MS/MS:* To tune the mass spectrometer Tuning Mix ES-TOF (Agilent Technologies) was directly infused at a 3 μL min^-1^ flow rate. To calibrate the mass spectrometer, lock mass internal calibration used a wick saturated with hexakis (1H,1H,3H-tetrafluoropropoxy) phosphazene ions (Synquest Laboratories, m/z 922.0098) located within the source to constantly infuse the calibrant into the instrument. Samples were introduced by a ThermoScientific UltraMate 3000 Dionex UPLC using a 30 μL injection volume. Methanol extracts were separated using a Kinetex 2.6 μm C18 (30 x 2.10 mm) UPLC column. MS spectra in the mass range *m/z* 50 – 2000 were acquired and the top ten most intense ions in a particular scan were fragmented using collision induced dissociation at 35 *e*V for +1 ions and 25 eV for +2 ions in the collision cell.

*Molecular Feature Table Generation:* The parameters of the call were: a signal to noise threshold of 5, correlation coefficient threshold of 0.7, a minimum compound length of 8 spectra, and a smoothing width of 2. The bucket table for this analysis was generated on all 198 reef samples using the time of the chromatogram run from 60 sec to 570 seconds and a window of *m/z*150 to 1500 using the Bruker Profile Analysis software version 2.1 build 282 (64-bit). The advanced bucketing feature was used including the time alignment and the value count of the bucket was greater than 20 samples.

*Molecular Networking*: All LC-MS/MS data collected was converted into the .mzXML format, using Bruker DataAnalysis software v4.1 in a batch process after applying the lock mass calibration. The coral networks used the following settings: parent mass tolerance: 0.5 Da; ion tolerance: 0.5 Da; cosine cutoff: 0.65; minimum matched peaks: 2; minimum cluster size: 2. Automatic identification against the GNPS spectral library used the following settings: library search minimum matched peaks: 4; score threshold: 0.7. Both group mapping and attribute mapping were used to enhance visualization of these molecular networks within Cytoscape (v.2.8.1). Edge widths were used to indicate cosine similarity where the thicker the edge the higher the cosine score. Color and size of the nodes was used to visually assist network analysis. Annotation of metabolites through GNPS was done by searching all spectra against the GNPS libraries with the following parameters: minimum matched peaks of 4, cosine score of 0.75 and a minimum peak intensity of 200. Hits to GNPS were further screened for those with a mass difference of less than 1*m/z* to be incorporated into the putatively annotated compound list (Table S2).

*Metatranscriptome Generation and Analysis*: RNA quality and concentration were quantified using Agilent RNA 6000 Pico Kit on the Agilent Bioanalyzer (Agilent technologies). RNA samples were depleted of rRNA using Ribo-Zero Gold Epidemiology rRNA removal kit (Illumina) and RNA fragments >200nt were purified using RNA Clean & Concentrator Kit (Zymo Research). SortMeRNA v. 2.0 was used to partition reads into rRNA and non-rRNA bins [3]. Transcriptome read and quality data is available in table S3.

1. Watrous, J. et al. 2012 Mass spectral molecular networking of living microbial colonies. *Proc. Natl. Acad. Sci. U. S. A.* **109**, 1743–52. (doi:10.1073/pnas.1203689109)

2. Vázquez-Baeza, Y., Pirrung, M., Gonzalez, A. & Knight, R. 2013 EMPeror: a tool for visualizing high-throughput microbial community data. *Gigascience* **2**, 16. (doi:10.1186/2047-217X-2-16)

3. Kopylova, E., Noé, L. & Touzet, H. 2012 SortMeRNA: fast and accurate filtering of ribosomal RNAs in metatranscriptomic data. *Bioinformatics* **28**, 3211–7. (doi:10.1093/bioinformatics/bts611)
