# Supplementary material for: Metabolomics of reef benthic interactions reveals a bioactive lipid involved in coral defence
Source: Proc Biol Sci. 2016 Apr 27;283(1829):20160469. doi: 10.1098/rspb.2016.0469 (PMC4855392; doi:10.1098/rspb.2016.0469)
Supplement: Supplementary table captions [file rspb20160469supp3.docx]

Table S1. Sample information of metabolome and transcriptomes from SLIs.

Table S2. Normalized abundance and standard deviation of Lyso-PAF and PAF forms in coral data.

Table S3. Putatively annotated metabolites from GNPS library search. Prevalence of the molecules in the different holobiont metabolomes and spectral alignment scores are shown.

Table S4. BLASTP results between human isoforms of *LysoPAF-AT*, *PLA2* and PAFAH against *Acropora digitifera* genome.

Table S5. Transcriptome raw read and quality data.
